# Machine learning identified novel players in lipid metabolism, endosomal trafficking, and iron metabolism of the ALS spinal cord

**DOI:** 10.1038/s41598-024-81315-z

**Published:** 2025-01-10

**Authors:** Jack Cheng, Bor-Tsang Wu, Hsin-Ping Liu, Wei-Yong Lin

**Affiliations:** 1 Graduate Institute of Integrated Medicine, College of Chinese Medicine, China Medical University, Taichung, 40402 Taiwan; 2 Department of Medical Research, China Medical University Hospital, Taichung, 40447 Taiwan; 3 Department of Senior Citizen Service Management, National Taichung University of Science and Technology, Taichung, 40343 Taiwan; 4 Graduate Institute of Acupuncture Science, College of Chinese Medicine, China Medical University, Taichung, 40402 Taiwan

**Keywords:** ALS, Spinal cord, Machine learning, RNA-seq, Amyotrophic lateral sclerosis, Machine learning

## Abstract

Amyotrophic lateral sclerosis (ALS) is a fatal neurodegenerative disease affecting motor neurons. Although genes causing familial cases have been identified, those of sporadic ALS, which occupies the majority of patients, are still elusive. In this study, we adopted machine learning to build binary classifiers based on the New York Genome Center (NYGC) ALS Consortium’s RNA-seq data of the postmortem spinal cord of ALS and non-neurological disease control. The accuracy of the classifiers was greater than 83% and 77% for the training set and the unseen test set, respectively. The classifiers contained 114 genes. Among them, 41 genes have been reported in previous ALS studies, and others are novel in this field. These genes are involved in mitochondrial respiration, lipid metabolism, endosomal trafficking, and iron metabolism, which may promote the progression of ALS pathology.

## Introduction

Amyotrophic lateral sclerosis (ALS) is a fatal neurodegenerative condition that progresses over time, marked by the deterioration of both upper and lower motor neurons that regulate voluntary movements through the corticospinal tract^1^. While the majority of patients experience the onset of the disease in middle age, there exists significant clinical diversity in symptom initiation and the rate of disease advancement leading up to mortality^2^. Approximately 5–10% of ALS cases are hereditary, with familial instances, and the remaining cases are considered sporadic^3^. Although mutations in TDP-43, C9orf72, SOD1, TARDBP, FUS, NEK1, TBK1, and KIF5A have been identified in familial ALS population^3^, importantly, a significant number of non-familial ALS, i.e., the majority of ALS cases, i.e., the sporadic ALS, lack clear causative genetics.

The New York Genome Center (NYGC) ALS Consortium is a cooperation of 42 global institutes aiming to collect genetic information from several thousand samples to tackle ALS-causing genetics, which are of moderate impact and relatively rare in the population^4^. Since 2018, ALS research has been advanced based on the data collected by the ALS Consortium, as well as Project MinE^5^, including KIF5A^6^, DNAJC7^7^, miR-218^8^, STMN2^9^, UNC13A^10^, IL18RAP^11^, VPS35^12^, and ATXN3^13^. However, the genetics underlying several clinical characteristics of ALS, including dysregulated energy metabolism^14^, lipid metabolism^15^, iron metabolism^16^, and intracellular transport, are still elusive.

One of the main difficulties in understanding and developing treatments for neurodegenerative diseases arises from the genetic heterogeneity present in these conditions^17^. This diversity in genetic factors can challenge the assumptions underlying traditional statistical methods like the T-test or ANOVA, which rely on normally distributed, independent samples and equal variance among groups^18^. When these assumptions are not met, the validity of such statistical approaches may be compromised. In contrast, machine learning classifiers are capable of identifying predictive patterns without needing to adhere to these assumptions^19^. For this reason, we have suggested that machine learning could be an effective supplementary method for studying diseases characterized by genetic heterogeneity. In fact, we have successfully employed machine learning techniques in our previous research on neurodegenerative conditions such as Alzheimer’s disease^20^ and Huntington’s disease^21^.

To uncover the ALS genetics, we applied machine learning on the ALS Consortium’s RNA-seq dataset of the postmortem spinal cord of ALS and non-neurological disease control in this study, and we report novel genes that may partly explain several clinical characteristics of ALS, such as dysregulated energy metabolism, lipid metabolism, iron metabolism, and intracellular transport.

## Results

**Fig. 1:**
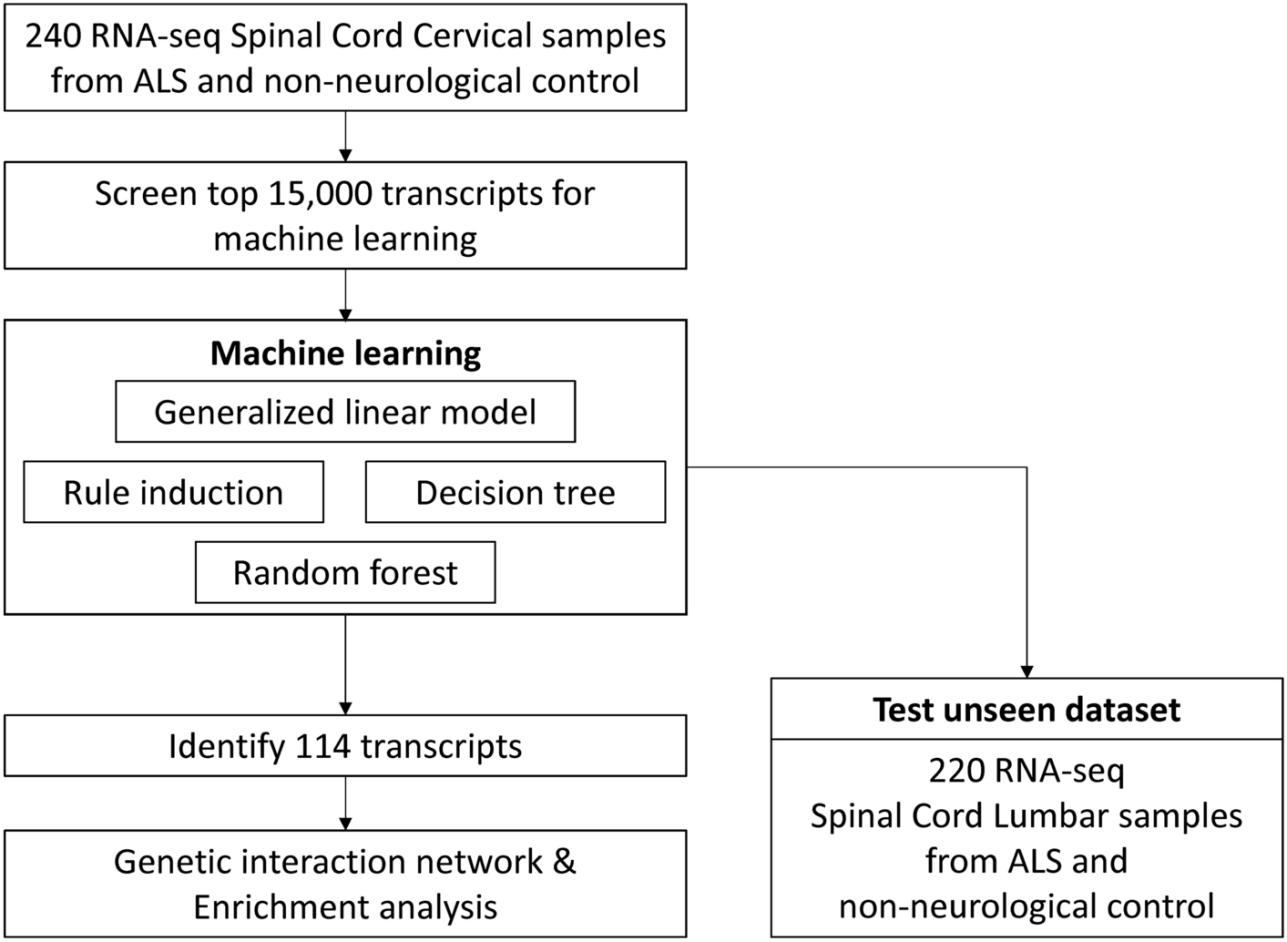
Workflow of this study. Each block represents one step in the workflow, and arrows represent the direction of information flow. The first step is retrieving the ALS RNA-seq dataset. The second step is data cleansing. The third step is building ML classifiers using the training dataset. Two divergent steps follow: the ML model validation using the unseen dataset (the right branch), and the identifying of transcripts (the left branch). The final step is enrichment analysis and interaction network.

**Fig. 2:**
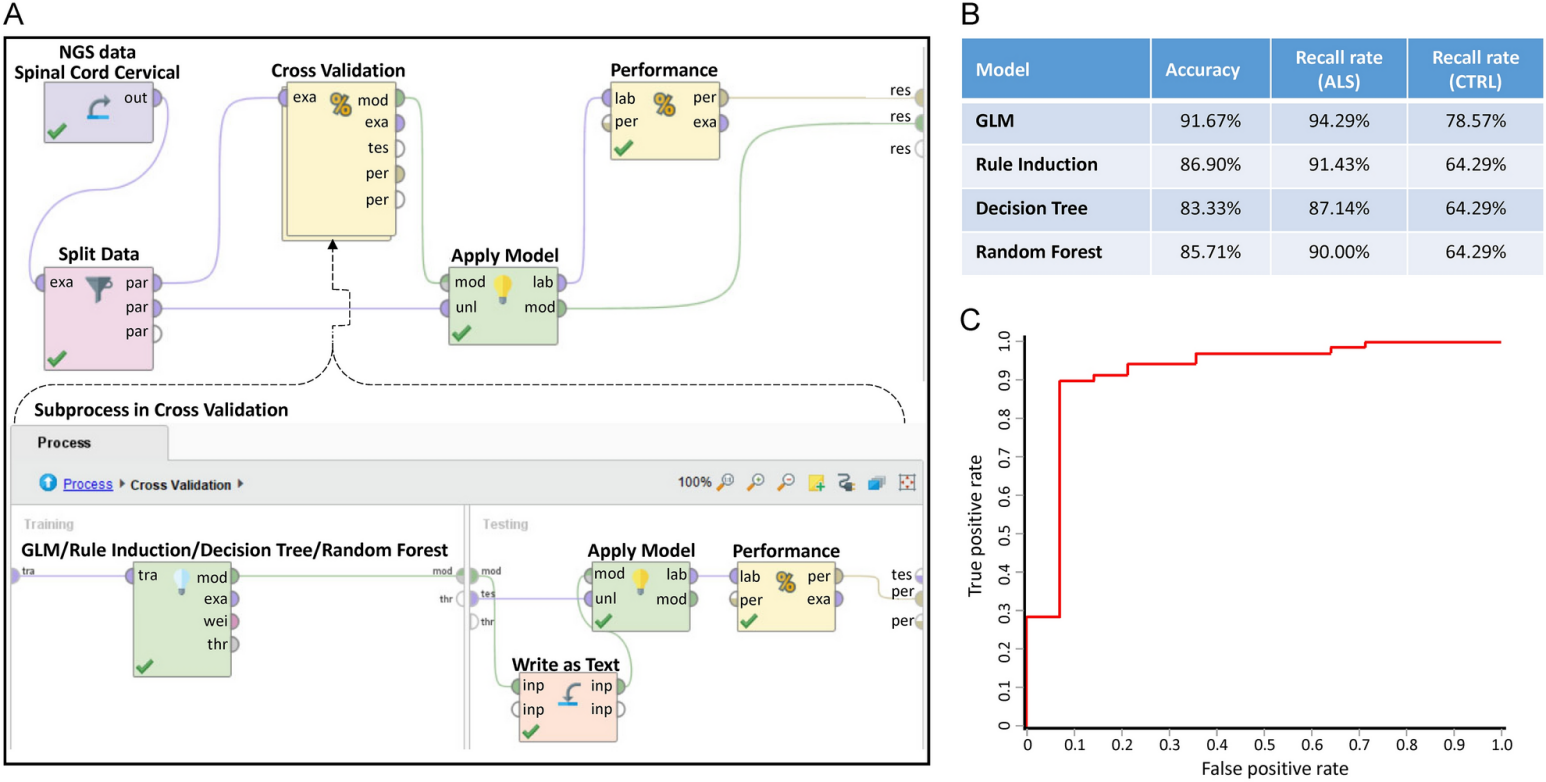
The building and performance of the binary classifiers of ALS. (**A**) The program setup for building binary classifiers of ALS. The subprocess inside the Cross-validation process is shown in the lower part. (**B**) The performance of the four established models. (**C**) ROC curve of the established binary classifier of ALS. This section focuses on the construction and evaluation of the binary classifiers used to distinguish between ALS and control samples. The process of building these classifiers involved several steps to ensure accuracy and robustness. In Figure A, we illustrate the overall program setup that was used for the creation of the binary classifiers targeting ALS. This diagram outlines the key components involved in developing the models, starting with data preprocessing, followed by feature selection and the subsequent model training. In (**B**), we present the performance metrics of the four different models that were established during the study. These models employed distinct machine learning algorithms, each optimized for binary classification tasks. The performance evaluation focused on key metrics such as accuracy and recall, all of which provided a comprehensive assessment of how well the classifiers were able to distinguish between ALS and control samples. (**C**) features the receiver operating characteristic (ROC) curve of the established binary classifier for ALS. The ROC curve is a crucial graphical representation of the classifier’s performance across different threshold settings. It plots the true positive rate (sensitivity) against the false positive rate (1-specificity) to evaluate the trade-offs between these two measures. A higher area under the ROC curve (AUC) indicates a better-performing model.

To better understand the pathological mechanisms in the ALS spinal cord that had previously been identified, we utilized RNA sequencing data from 240 cervical spinal cord samples, which included both ALS patients and non-neurological disease controls. This dataset served as the input for machine learning models designed to create binary classifiers capable of distinguishing between ALS and control samples. The entire workflow for building these classifiers is illustrated in Fig. 1. In our analysis, we employed four different machine learning algorithms: "Generalized Linear Model" (GLM), "Rule Induction," "Decision Tree," and "Random Forest." Each of these algorithms was carefully programmed, and their respective processes are depicted in Fig. 2A. To evaluate the robustness of the models, we conducted cross-validation, with the results for this process displayed in Fig. 2B.

All four algorithms performed remarkably well, achieving an overall accuracy—defined as the total true positive rate—of more than 80%. This high accuracy suggests that the binary classifiers were able to effectively differentiate between the ALS and control samples. However, we did observe a bias in the recall rates of the models. Specifically, the recall rate for ALS predictions was higher compared to the recall rate for control predictions. This discrepancy in recall rates could likely be attributed to an imbalance in the sample sizes: the dataset contained 199 ALS samples compared to only 41 control samples, resulting in an approximately 5:1 ratio between ALS and control groups. Such an imbalance in the data could skew the classifiers to favor ALS predictions over control predictions.

Despite this bias, the performance of the classifiers remained robust, particularly for the GLM algorithm. As shown in Fig. 2C, the receiver operating characteristic (ROC) curve for GLM indicates that the model maintained strong performance, even when operating at lower confidence thresholds. This suggests that GLM is able to achieve good predictive accuracy while maintaining flexibility in its decision-making process, making it a reliable tool for identifying ALS-related patterns within the RNA-seq data.

**Fig. 3:**
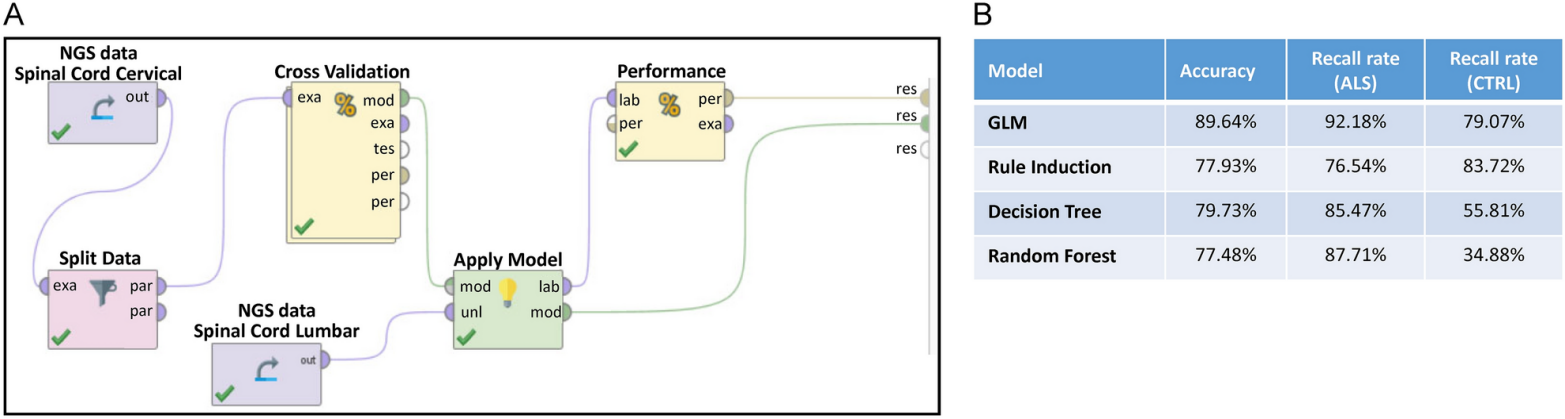
The validation of the binary classifiers of ALS using an unseen dataset. (**A**) The program setup for validating the binary classifier of ALS. (**B**) The performance of the four established models. This figure details the validation process of the binary classifiers designed to distinguish ALS samples from controls, using an independent dataset that was not included in the model-building phase. This “unseen dataset” is critical for evaluating how well the classifiers generalize to new data and ensuring that their performance holds up outside the training environment. The unseen dataset contains samples that the models have not encountered before, which allows us to assess the true predictive power of the classifiers in real-world scenarios. This validation process is a crucial step in confirming that the binary classifiers are not overfitted to the training data and can reliably generalize to new, unseen datasets.

To further assess the effectiveness of our trained classifiers, we next applied them to an unseen dataset, comprising RNA sequencing data from 222 lumbar spinal cord samples. This dataset included both ALS patients and non-neurological disease controls, and it allowed us to independently validate the performance of the classifiers we had previously developed. The programming workflow for this validation process is illustrated in Fig. 3A, while the performance metrics of the classifiers are summarized in Fig. 3B.

Across all four machine learning algorithms, the classifiers maintained a strong level of accuracy, exceeding 77% in all cases. This high accuracy suggests that the models were able to generalize well to new data and reliably distinguish between ALS and control samples. Furthermore, the recall rate for ALS predictions was consistently high, with all classifiers achieving a recall rate above 76%. This indicates that the classifiers were effective in correctly identifying ALS cases from the new dataset, which is crucial for ensuring that the models can be used to detect ALS with confidence.

However, the performance of the classifiers was more variable when it came to predicting control samples. The recall rate for controls ranged widely, from as low as 34% to as high as 83%, depending on the algorithm. This variation in recall rates for the control group may be due to several factors. The consistently high recall rate for ALS predictions suggests that the pathological genetic features associated with ALS are relatively similar across different parts of the spinal cord. In other words, the genetic patterns found in the cervical spinal cord, which were used to train the classifiers, appear to also be present in the lumbar spinal cord, making it easier for the models to detect ALS regardless of spinal cord region.

On the other hand, the lower recall rates for control samples in some classifiers could indicate that there are slight differences in the genetic background of the non-neurological disease controls between the cervical and lumbar regions of the spinal cord. These subtle genetic variations might make it more challenging for the classifiers to accurately identify control samples in the lumbar dataset, leading to a wider range of recall performance. This finding highlights the possibility that different parts of the spinal cord may exhibit distinct genetic characteristics, especially in non-neurological conditions, and it suggests that further refinement of the classifiers may be necessary to improve their performance in detecting control samples across diverse spinal cord regions.

**Fig. 4:**
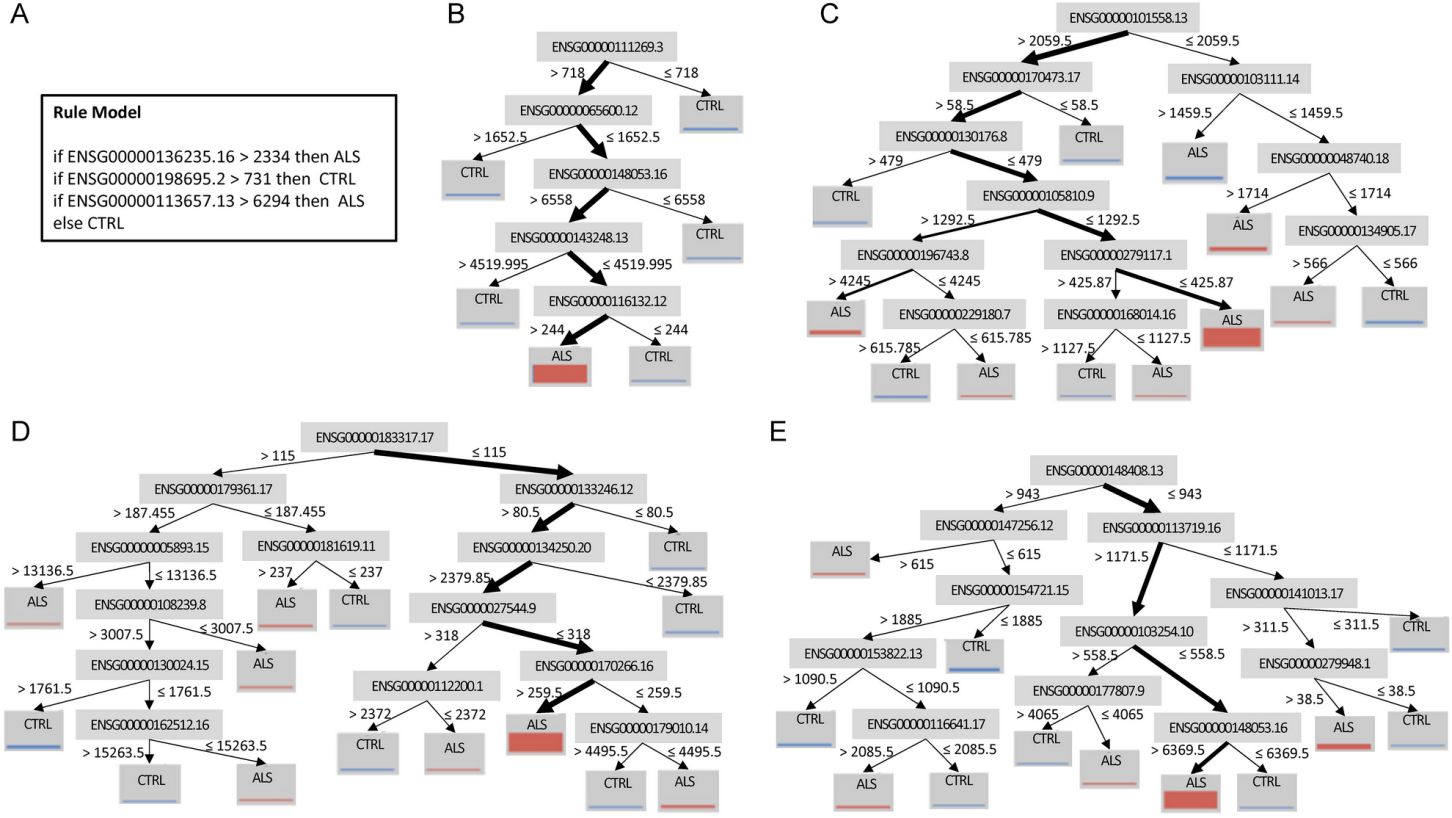
The binary classifiers of ALS. (**A**) Rule induction. Rule induction systematically identifies patterns in the data and constructs a set of "if–then" rules that can be used to make predictions. For ALS classification, the rule induction classifier determines a series of logical conditions that differentiate ALS samples from control samples based on gene expression patterns. (**B**) Decision tree. The decision tree classifier uses the gene expression data to form a tree-like structure where each node represents a decision about a specific gene. The branches of the tree lead to different classifications—either ALS or control—based on the outcomes of these decisions. (**C**–**E**) Decision trees from Random forest. Each individual decision tree in a Random Forest is constructed from a random subset of the data, and the final classification is determined by aggregating the predictions from all the trees. In (**B**–**E**), the judgment criteria are noted near the splitting arrows, and the thickness of the arrows roughly represents the fraction of samples that fall in this criterion.

The classifiers that were developed and trained during our study are displayed in various figures and supplementary tables. Specifically, Fig. 4A illustrates the Rule Induction classifier, Fig. 4B shows the Decision Tree classifier, and Fig. 4C–E depict the Random Forest classifiers. Additionally, the Generalized Linear Model (GLM) is provided in Supplementary Table 1. Together, these four classifiers collectively identified 114 genes, which are listed in Supplementary Table 2. These genes represent key distinguishing features between ALS spinal cord samples and those from non-neurological disease controls.

**Fig. 5:**
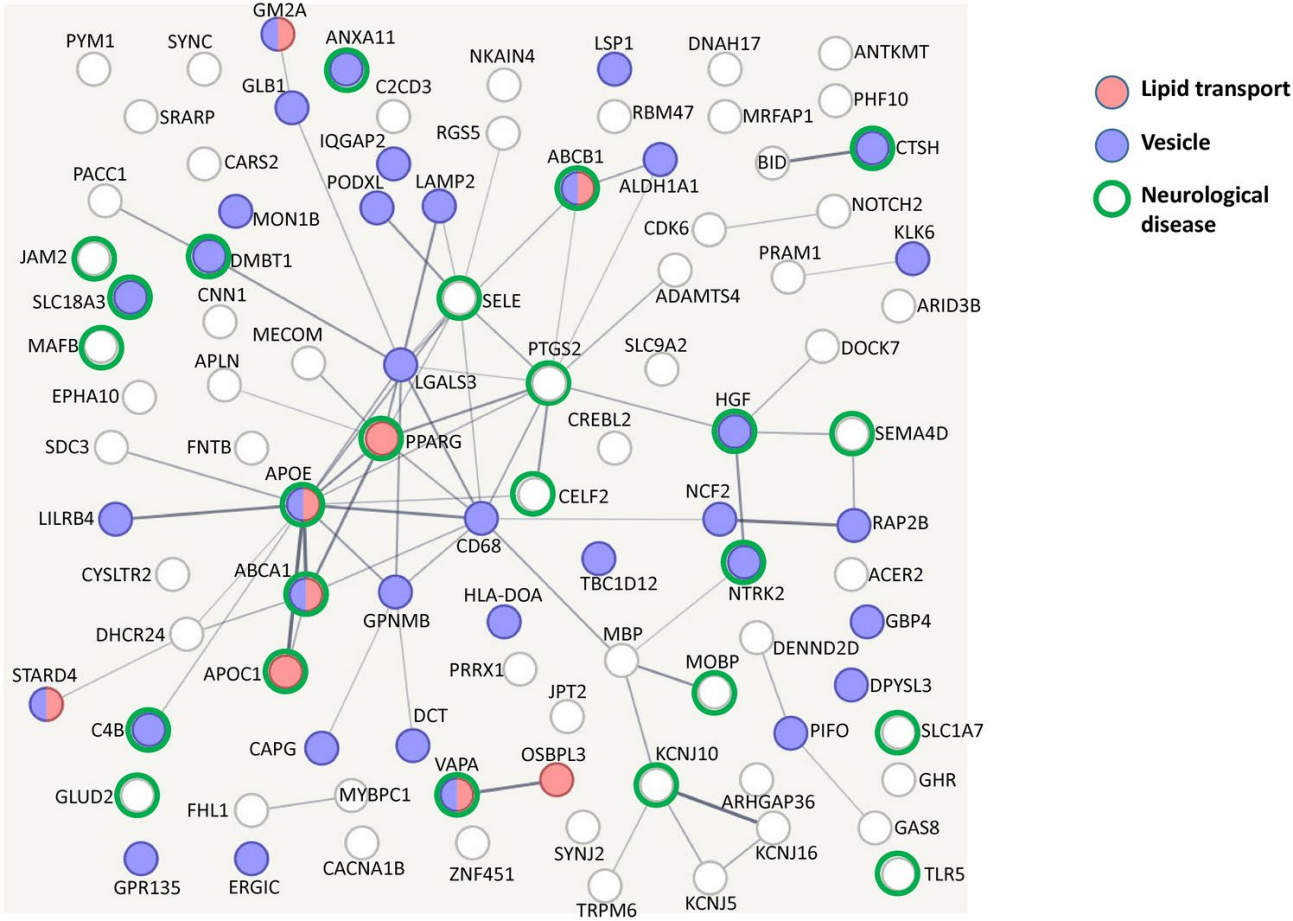
Interaction network of the genes used in the binary classifiers of ALS. The colors of the nodes denote their enriched biological processes. The thickness of the edges denotes the confidence of the connection between nodes.

What is particularly noteworthy about these 114 genes is their emphasis on the composition of vesicles and lipid transport as the primary factors differentiating ALS from controls, as visualized in Fig. 5. This finding is important because it suggests that disruptions in vesicle formation and lipid movement may play a central role in the pathological mechanisms of ALS, offering a potential area for further investigation into the disease’s molecular underpinnings.

Out of the 114 genes identified by the classifiers, 41 had been previously reported in other ALS research, as shown in Table 1. This overlap reinforces the relevance of these genes to ALS and suggests that our machine-learning approach was successful in pinpointing key genetic markers that have already been associated with the disease. However, the study also revealed 73 genes that are novel to ALS research, meaning they had not been reported in prior studies. This is a significant finding, as it expands our understanding of the genetic factors involved in ALS. Among these 73 newly identified genes, eight have been linked to other neurological diseases, as outlined in Table 2. This connection suggests that some genetic pathways may be shared across multiple neurological disorders, opening up potential avenues for cross-disease research.

**Table 1:** Identified genes that have been reported in previous ALS studies. The first column contains the Ensembl transcript ID. The second column contains the gene symbol of the transcript. The third column shows the involvement of the transcript in the trained ML classifier. The fourth and fifth columns show the type of previous study and reference that identified the involvement of the gene in ALS.

Accession	Symbol	Used in Classifiers	ALS study	References
ENSG00000165092.13	ALDH1A1	GLM	ALS association	^22^
ENSG00000130208.9	APOC1	GLM	ALS association	^23^
ENSG00000224389.9	C4B	GLM	ALS association	^24^
ENSG00000042493.16	CAPG	GLM	ALS association	^25^
ENSG00000103811.16	CTSH	GLM	ALS association	^26^
ENSG00000187775.16	DNAH17	GLM	ALS association	^27^
ENSG00000268388.5	FENDRR	GLM	ALS association	^28^
ENSG00000170266.16	GLB1	Random forest	ALS association	^29^
ENSG00000204252.14	HLA-DOA	GLM	ALS association	^30^
ENSG00000145703.16	IQGAP2	GLM	ALS association	^13^
ENSG00000154721.15	JAM2	Random forest	ALS association	^31^
ENSG00000131981.16	LGALS3	GLM	ALS association	^32^
ENSG00000186818.12	LILRB4	GLM	ALS association	^33^
ENSG00000197971.15	MBP	GLM	ALS association	^34^
ENSG00000116701.14	NCF2	GLM	ALS association	^35^
ENSG00000134250.20	NOTCH2	Random forest	ALS association	^36^
ENSG00000073756.12	PTGS2	GLM	ALS association	^37^
ENSG00000187714.7	SLC18A3	GLM	ALS association	^38^
ENSG00000162383.12	SLC1A7	GLM	ALS association	^39^
ENSG00000286159.1	Antisense To PREX1	GLM	ALS GWAS	^40^
ENSG00000122359.18	ANXA11	GLM	ALS GWAS	^41^
ENSG00000130203.10	APOE	GLM	ALS GWAS	^42^
ENSG00000116133.13	DHCR24	GLM	ALS GWAS	^43^
ENSG00000113657.13	DPYSL3	Rule induction	ALS GWAS	^44^
ENSG00000113719.16	ERGIC1	Random forest	ALS GWAS	^45^
ENSG00000196743.8	GM2A	Random forest	ALS GWAS	^46^
ENSG00000168314.17	MOBP	GLM	ALS GWAS	^47^
ENSG00000132170.21	PPARG	GLM	ALS GWAS	^48^
ENSG00000165029.16	ABCA1	GLM	ALS mechanism	^49^
ENSG00000085563.14	ABCB1	GLM	ALS mechanism	^50^
ENSG00000158859.10	ADAMTS4	GLM	ALS mechanism	^51^
ENSG00000147256.12	ARHGAP36	Random forest	ALS mechanism	^52^
ENSG00000129226.14	CD68	GLM	ALS mechanism	^53^
ENSG00000105810.9	CDK6	Random forest	ALS mechanism	^54^
ENSG00000187908.18	DMBT1	GLM	ALS mechanism	^55^
ENSG00000136235.16	GPNMB	GLM, Rule induction	ALS mechanism	^56^
ENSG00000019991.17	HGF	GLM	ALS mechanism	^57^
ENSG00000177807.9	KCNJ10	Random forest	ALS mechanism	^58^
ENSG00000148053.16	NTRK2	Decision tree, Random forest	ALS mechanism	^59^
ENSG00000187764.11	SEMA4D	GLM	ALS mechanism	^60^
ENSG00000101558.13	VAPA	Random forest	ALS mechanism	^61^

**Table 2:** Identified genes that are related to other neurological diseases. The first column contains the Ensembl transcript ID. The second column contains the gene symbol of the transcript. The third column shows the involvement of the transcript in the trained ML classifier. The fourth and fifth columns show the type of diseases and references that identified the involvement of the gene.

Accession	Symbol	Used in classifiers	Diseases	References
ENSG00000048740.18	CELF2	Random forest	Encephalopathy	^62^
ENSG00000182890.4	GLUD2	GLM	Parkinson’s disease	^63^
ENSG00000167755.15	KLK6	GLM	Hydrocephalus	^64^
ENSG00000204103.4	MAFB	GLM	Alzheimer’s disease	^65^
ENSG00000198763.3	MT-ND2	GLM	Leigh syndrome	^66^
ENSG00000198695.2	MT-ND6	GLM, rule induction	Leigh syndrome	^67^
ENSG00000007908.16	SELE	GLM	Brain ischemia	^68^
ENSG00000187554.13	TLR5	GLM	Brain ischemia	^69^

Additionally, 21 of the novel genes are involved in vesicle formation, ion channel function, or lipid transport, as detailed in Table 3. These genes are particularly intriguing because they point to new areas of investigation in ALS research. Their involvement in key cellular processes that are essential for neuron function and signaling suggests that further exploration of these pathways could yield valuable insights into how ALS develops at the molecular level. Given that these 21 genes have not been previously associated with ALS, their discovery offers a fresh perspective on the disease’s biology and could help to identify new therapeutic targets aimed at addressing these specific mechanisms.

**Table 3:** Identified genes that are involved in vesicle, ion channel, or lipid transportation. The first column contains the Ensembl transcript ID. The second column contains the gene symbol of the transcript. The third column shows the involvement of the transcript in the trained ML classifier. The fourth column shows the biological pathway that the gene is involved in.

Accession	Symbol	Used in classifiers	Involves in
ENSG00000177076.6	ACER2	GLM	Lipid metabolism
ENSG00000111269.3	CREBL2	Decision tree	Lipid genesis
ENSG00000070882.13	OSBPL3	GLM	Lipid transport
ENSG00000164211.13	STARD4	GLM	Lipid transport/vesicle
ENSG00000080166.16	DCT	GLM	Vesicle
ENSG00000162654.9	GBP4	GLM	Vesicle
ENSG00000181619.11	GPR135	Random forest	Vesicle
ENSG00000005893.15	LAMP2	Random forest	Vesicle
ENSG00000130592.15	LSP1	GLM	Vesicle
ENSG00000103111.14	MON1B	Random forest	Vesicle
ENSG00000173947.14	PIFO	GLM	Vesicle
ENSG00000128567.17	PODXL	GLM	Vesicle
ENSG00000181467.4	RAP2B	GLM	Vesicle
ENSG00000078269.15	SYNJ2	GLM	Vesicle endocytosis
ENSG00000108239.8	TBC1D12	Random forest	Vesicle
ENSG00000148408.13	CACNA1B	Random forest	Calcium channel
ENSG00000153822.13	KCNJ16	Random forest	Potassium channel
ENSG00000120457.12	KCNJ5	GLM	Potassium channel
ENSG00000101198.15	NKAIN4	GLM	Sodium–potassium pump
ENSG00000065600.12	PACC1	Decision tree	Chloride channel
ENSG00000115616.2	SLC9A2	GLM	Sodium antiporter

To evaluate whether age, sex, and genetic variations correspond to specific RNA expression patterns in ALS, we re-run the analysis using age, sex, and the GGGGCC repeat size of C9orf72 or CAG repeat size of ATXN2 as the Machine-learning “labels” according to the clinical information of ALS samples.

For age as the ML label, the two strategies shown in Supplementary Fig. 1 were used. In the first strategy (the left flow in Supplementary Fig. 1), the label age was treated as continuous numbers, and the ML model of linear regression was trained using RNA expression. The resulting root-mean-square deviation (RMSD) of the regression model was 9.459 ± 3.604 years, which is not significantly different from the standard deviation of ALS samples of 10.05 years. This means the linear regression model could not effectively predict the age of ALS samples. In the second strategy (the right flow in Supplementary Fig. 1), the label age was treated as discrete data, i.e., label = 1 if age < 55, else label = 0, and the trained classification models were GLM, decision tree, random forest, and rule induction. The resulting AUC of the ROC curve for GLM, decision tree, random forest, and rule induction were 0.684, 0.500, 0.577, and 0.573, respectively, not much better than a 50% chance of flipping a coin. Meanwhile, the recall rate of age-under-55 ALS samples was 12.5%, 31.25%, 18.75%, and 12.5%, respectively. This means that trained classification models could not effectively predict the age of ALS samples. In sum, neither strategy could predict the age of ALS samples, i.e., we cannot claim the correlation between the age and the RNA expression pattern of ALS samples.

**Fig. 6:**
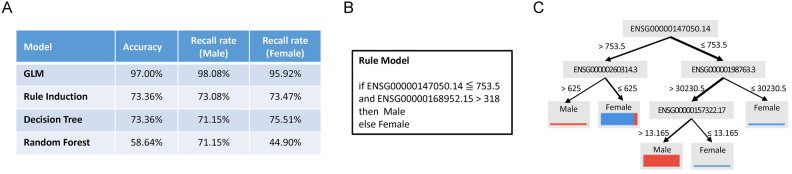
The binary classifiers of the sex of ALS. (**A**) The performance of the four established models. (**B**) Rule induction. (**C**) Decision tree.

For sex as the ML label, i.e., the label for male = 1 and female = 0, we conducted two rounds of ML as shown in Supplementary Fig. 2. In the first round (the left flow in Supplementary Fig. 2), all RNA of ALS samples in the dataset were kept for the training of prediction models; as a result, we got 100% recall rate for both sexes. This result met our anticipation since Y chromosome genes were included. Therefore, in the second round (the right flow in Supplementary Fig. 2), Y chromosome genes were excluded from the training dataset, and the recall rate for both sexes was > 70% for three models (see Fig. 6A for detail, 6B for the rule induction, 6C for the decision tree, and Supplementary Table 6 for the GLM models). Interestingly, there is a common gene in the three models: ENSG00000147050.14 (KDM6A, lysine demethylase 6A).

For genetic variation as the ML label, we set the label = 1 for those ALS samples carrying more than 30 GGGGCC repeats in C9ORF72 or intermediate (i.e., 30–33,) CAG repeats in ATXN2, and label = 0 for those ALS samples who did not meet the previous criteria. Using the workflow shown in Supplementary Fig. 3, the resulting AUC of the ROC curve for GLM, rule induction, decision tree, and random forest, were 0.508, 0.558, 0.508, and 0.454, respectively. Meanwhile, the recall rate of ALS samples carrying genetic mutation was 5.00%, 15.00%, 25.00%, and 30.00%, respectively. This means that trained classification models could not effectively predict the genetic mutation of ALS samples, i.e., we cannot claim the correlation between the genetic mutation and the RNA expression pattern of ALS samples.

## Discussion

In this study, we generated several ALS-Control classifiers using an RNA-seq dataset from the cervical spinal cord and used the counterpart from the lumbar spinal cord as the unseen dataset for validation. The accuracy of classifiers was higher than 83% for the cross-validation during model building and 77% for the unseen dataset, which not only justifies the performance of the classifiers but also indicates the similarity of ALS transcriptomic signatures between different parts of the spinal cord. The relevance to previous ALS studies and the biological meaning of the novel findings are discussed below.

In the generated ALS- Control classifiers, 41 genes have been reported in previous ALS studies (Table 1), which cover different study types, including association studies, genome-wide association studies (GWAS), and mechanism research. We will not discuss these genes, but we have provided a reference list in Table 1 if more detail is needed. Notably, the rediscovery of the previously identified ALS genes strengthens the reliability of our study. In addition to those rediscovered ones, we identified 73 genes novel to ALS research. Among the novel genes, those eight genes in Table 2 have been mentioned in other neurological diseases, and those 21 genes in Table 3 involve critical biological functions of the spinal cord. Some of them may passively reflect the biological environment of ALS, but some may actively make progress in ALS pathology. We shall focus our discussion on the later part, which will be divided into four groups according to their biological functions, including mitochondrial respiration, lipid metabolism, endosomal trafficking, and ion channel.

The mitochondrial NADH dehydrogenase 2 (mt-ND2) and NADH dehydrogenase 6 (mt-ND6) are subunits of the NADH dehydrogenase, which is the largest electron transport chain complex in the mitochondrial inner membrane and responsible for mitochondrial ATP synthesis^70^. As shown in the Rule Induction classifier (Fig. 4A), a sample is classified as a non-ALS control if the expression of ENSG00000198695.2, i.e., mt-ND6, is greater than a certain level. This criterion indicates that the quantity of the NADH dehydrogenase subunit is fewer for the ALS spinal cord. Importantly, mt-ND6 is essential for the assembly of the membrane arm of the NADH dehydrogenase and is indispensable for mitochondrial respiratory function^70^. Interestingly, disrupted TCA cycle^71^ and increased glycolysis^72^ have been reported in ALS models. Thus, insufficient levels of mt-ND6 may directly limit the efficiency of mitochondrial respiration and indirectly force the utilization of glycolysis to fulfill the energy demand.

Altered lipid metabolism has been identified in animal models^29,73^ and cohorts^74^ of ALS. Interestingly, several rediscovered ALS genes in previous studies are relevant to lipid transport (Fig. 5), including APOE, APOC1, ABCA1, ABCB1, GM2A, PPARG, and VAPA. The novel ones are oxysterol-binding protein-related protein 3 (OSBPL3), StAR-related lipid transfer protein 4 (STARD4), and sphingolipid long chain base-responsive protein LSP1 (LSP1). OSBPL3 is located in the membrane contact site between plasma and endoplasmic reticulum (ER) membranes and forms a complex with VAPA^75^. OSBPL3 regulates plasma membrane phosphatidylinositol 4-phosphate (PI4P) levels and Ca^2^^+^ entry by exchanging phosphatidylcholine^76^. STARD4 regulates intracellular cholesteryl ester formation, sterol transport to the ER, and SREBP-2-mediated sterol sensing by SCAP/SREBP-2^77^. LSP1 localizes at eisosomes and participates in lipid endocytosis^78^. The dysregulation of OSBPL3, STARD4, and LSP1 may exacerbate the altered lipid metabolism in ALS. In sum, the lipid transport pathway plays an important biological role in ALS, particularly in relation to how it affects neurons and their ability to function properly. Lipids are essential for maintaining the structure and function of cell membranes, particularly in neurons. They also serve as a source of energy. Lipid metabolism disruptions have been linked to ALS, with many patients experiencing altered lipid profiles, including elevated cholesterol and triglycerides^15^. These genes may affect how lipids are transported within neurons, leading to cellular stress or degeneration. Dysregulation of these genes can impair the normal trafficking and metabolism of lipids, disrupting cellular energy balance and membrane integrity. Defects in lipid transport can lead to neuronal dysfunction and contribute to motor neuron death, a hallmark of ALS.

The synaptic vesicle plays a crucial role in the pathology of ALS, primarily through its involvement in neurotransmitter release and neuronal communication^79^. Synaptic vesicles are small sacs that store neurotransmitters, which are chemicals used for communication between neurons. During synaptic transmission, vesicles fuse with the presynaptic membrane, releasing neurotransmitters into the synaptic cleft. This allows for the activation of postsynaptic neurons, facilitating communication between neurons, including motor neurons. Importantly, motor neurons, with their long axons, depend heavily on the efficient transport of synaptic vesicles from the cell body to the synapse. Disruptions in axonal transport mechanisms, often associated with ALS^80^, affect the delivery of synaptic vesicles, leading to synaptic dysfunction and degeneration of motor neuron connections. Endosomal trafficking is critical in maintaining the proper function of neurons, in the context of targeted transportation and protein recycling in the extremely asymmetric and complex intracellular space of a neuron^81^. Interestingly, endosomal trafficking is disrupted by either C9ORF72^82^ or SOD1^83^ mutant in ALS. In this study, five identified genes involved in the endosome, including G-protein coupled receptor 135 (GPR135), Lysosome-associated membrane glycoprotein 2 (LAMP2), Vacuolar fusion protein MON1 homolog B (MON1B), Ras-related protein Rap-2b (RAP2B), and TBC1 domain family member 12 (TBC1D12). The dysregulation of these genes may contribute to the disruption of endosomal trafficking and promote neurodegeneration in ALS.

Abnormal accumulation of iron in CNS has been detected in neurodegenerative diseases, including ALS^84^. Iron level in the spinal cord is increased more than 1.5 fold in ALS^85^. Iron excess may induce oxidative stress^86^, ferroptosis^87^, and microglia activation^88^. In this study, we identified a proton-activated chloride channel (PACC1). PACC1 is a transmembrane protein that mediates the influx of chloride ions in response to extra-membrane acidic pH value^89^. Besides cellular membrane, PACC1 can translocate to endosomes and regulate transferrin receptor-mediated endocytosis^90^. As shown in Fig. 4B, lower PACC1, i.e., ENSG00000065600.12, predicts ALS, and according to the previous study^89^, PACC1 knockout results in increased transferrin uptake. Thus, the PACC1 down-regulation may promote neurodegeneration by mediating abnormal accumulation of iron in the spinal cord.

Men are generally more likely to develop ALS than women, particularly in younger age groups. However, this sex difference decreases with age. In older populations, the ratio between men and women diagnosed with ALS tends to even out^91,92^. Moreover, sporadic ALS (the most common form, making up 90–95% of cases) tends to occur more frequently in men, while familial ALS (accounting for 5–10% of cases) shows less of a sex disparity^91,93^. Although hormonal differences, especially related to estrogen, are considered a possible explanation for the sex disparity of ALS^94^, further investigations are needed to clarify this issue. In this study, we identified KDM6A as the common gene in sex classifiers of ALS, and a higher expression level of KDM6A predicts female ALS samples. KDM6A belongs to the family of histone demethylases, which modulate epigenetics during neurodevelopment and neurodegenerative diseases^95^. Interestingly, a previous study using microarray to probe blood RNA expression also identified higher expression levels of KDM6A in female than male ALS^96^. The exact role of KDM6A in ALS requires further investigation.

In conclusion, binary classifiers build by machine learning on spinal cord RNA-seq data successfully differentiate ALS and control samples. Besides, this study identified novel genes in mitochondrial respiration, lipid metabolism, endosomal trafficking, and iron metabolism, which may promote the progression of ALS pathology.

## Methods

### Source of NGS datasets

RNA-seq data of ALS and non-neurological control were retrieved from the Gene Expression Omnibus (GEO) database^97^ of the National Center for Biotechnology Information (NCBI) of the USA with the accession number GSE153960^9^, accessed on Sep 12th 2023, which contained RNA-seq data from the cervical and lumbar spinal cord, and the lumbar spinal cord samples were from the same cases/controls as the cervical spinal cord samples. For the development and cross-validation of the binary classifiers designed to differentiate between ALS and non-neurological conditions, we utilized data from 199 ALS samples and 41 non-neurological control samples derived from the cervical region of the spinal cord. This dataset provided the foundation for constructing the models and performing the necessary cross-validation to ensure the accuracy and reliability of the classifiers. Detailed information about this dataset, as well as the specifics of the model-building process, can be found in Supplementary Table 3. In addition to the training and validation performed on the cervical spinal cord dataset, we employed another independent set of data from the lumbar spinal cord to further test the generalizability of the classifiers. This unseen dataset consisted of RNA sequencing data from 179 ALS samples and 43 non-neurological control samples. By using this new dataset, we aimed to validate the performance of the classifiers on data that had not been used during the model training phase, ensuring that the classifiers could reliably predict ALS even when applied to samples from a different region of the spinal cord. This process of external validation helps to assess how well the classifiers can generalize to new data and different contexts, and the results of this validation, along with the details of the lumbar spinal cord dataset, are provided in Supplementary Table 4. By using distinct datasets from two different regions of the spinal cord—cervical for model building and cross-validation, and lumbar for independent testing—we were able to thoroughly evaluate the robustness and reliability of the classifiers. The inclusion of both regions ensures that the models are not overly specific to a single area of the spinal cord, increasing the likelihood that they will be applicable across different anatomical regions affected by ALS. This two-phase validation approach enhances the credibility of our findings, as it demonstrates that the models are capable of accurately predicting ALS across diverse sample sets, which is a critical step in advancing the potential for these classifiers to be used in broader clinical applications. The clinical information of ALS samples is listed in Supplementary Table 5.

### Data cleansing

In the process of building the binary classifiers, we used the field labeled "Sample id alt" to represent the unique identifier for each sample, which was referred to as "ID." This “ID” allowed us to track and differentiate between individual samples throughout the analysis. Meanwhile, the field labeled “Group” served as the "Label," which functioned as the target variable for the binary classification task. The “Label” distinguished between the two groups of interest—ALS and non-neurological control—and was the outcome the classifiers were trained to predict. For the actual features, or input variables, used to train the classifiers, we relied on the “ENSEMBL ID” of transcripts, which was designated as the "Regular Attribute." This means that each transcript, identified by its unique ENSEMBL ID, was used as a predictive feature in the machine learning models. The transcripts represent the genetic expression data from the samples, and these were the variables that the models analyzed to learn patterns associated with ALS or control groups. To focus the learning tasks on the most relevant data, we applied a filtering step to reduce the number of transcripts included in the analysis. Specifically, we retained only the top 15,000 transcripts with the highest average read counts across the dataset. By keeping only these top transcripts, we ensured that the models were trained on the most informative and reliable genetic data, as transcripts with higher read counts are generally more robust and less prone to noise or variability. This step was crucial for improving the efficiency and accuracy of the learning tasks, as it allowed the classifiers to focus on the most significant genetic signals that could differentiate between ALS and control samples.

### Machine learning

RapidMiner Studio version 9.5, running on a desktop PC with 16 GB RAM, was used to build and validate the binary classifiers of ALS. RNA-seq data of spinal cord cervical were split into 65% and 35% for model building and testing, respectively. Four algorithms were used, including the “Generalized Linear Model” (GLM), “Rule Induction”, “Decision Tree”, and “Random Forest”. The parameters are described as follows. Parameters of GLM: binomial family, IRLSM solver, use regularization, conduct lambda search, 47 lambdas, lambda min ratio of 0, use early stopping, 3 stopping rounds and stopping tolerance of 0.02.

Parameters of Rule Induction: criterion of information gain, sample ratio of 0.9, pureness of 0.9, and minimal prune benefit of 0.25.

Parameters of Decision Tree: criterion of gain ratio, with a maximal depth of 10, apply pruning with confidence of 0.1, apply prepruning with minimal gain of 0.01, minimal leaf size of 2, minimal split size of 4, and number of prepruning alternatives of 3.

Parameters of Random Forest: number of trees of 3, criterion of Gini index, maximal depth of 10, guess subset ratio, and voting strategy of confidence vote.

### Interaction network

String-db version 12.0^98^, accessed on Nov 20th, 2023, was used to generate the interaction network. The enrichment analysis was conducted using DAVID Bioinformatics Resources^99^, accessed on Nov 22nd, 2023. By utilizing String-db, we were able to visualize the potential relationships and interactions between different proteins, offering deeper insights into how these proteins might work together or influence one another in the context of ALS pathology. The use of such a resource significantly enhanced our ability to interpret the biological relevance of the identified genes, particularly in understanding how they may be functionally connected. In addition to building the interaction network, we performed an enrichment analysis to identify the biological pathways, functions, and processes that are overrepresented in the gene set. For this, we used the DAVID Bioinformatics Resources tool. Through this tool, we were able to explore which biological processes and molecular functions are significantly enriched in the genes identified in our study. By leveraging DAVID, we could link the identified genes to specific biological pathways, providing further context for their roles in ALS and other neurological conditions. This analysis enabled us to uncover patterns and commonalities among the genes, offering potential clues as to how genetic dysregulation might contribute to the progression of ALS.

## Supplementary Information

Supplementary Information 1.

Supplementary Information 2.

Supplementary Information 3.

## Data Availability

All data in this study are included in the supplementary data. The RNA-seq dataset can be accessed via the GEO database of the NCBI of the USA with the accession number GSE153960, with the website below. https://www.ncbi.nlm.nih.gov/geo/query/acc.cgi?acc=GSE153960.
