# Supplementary material for: Machine learning identified novel players in lipid metabolism, endosomal trafficking, and iron metabolism of the ALS spinal cord
Source: Sci Rep. 2025 Jan 10;15:1564. doi: 10.1038/s41598-024-81315-z (PMC11723943; doi:10.1038/s41598-024-81315-z)
Supplement: Supplementary file 1 — Supplementary Information 1. [file 41598_2024_81315_MOESM1_ESM.pptx]

## Slide 1
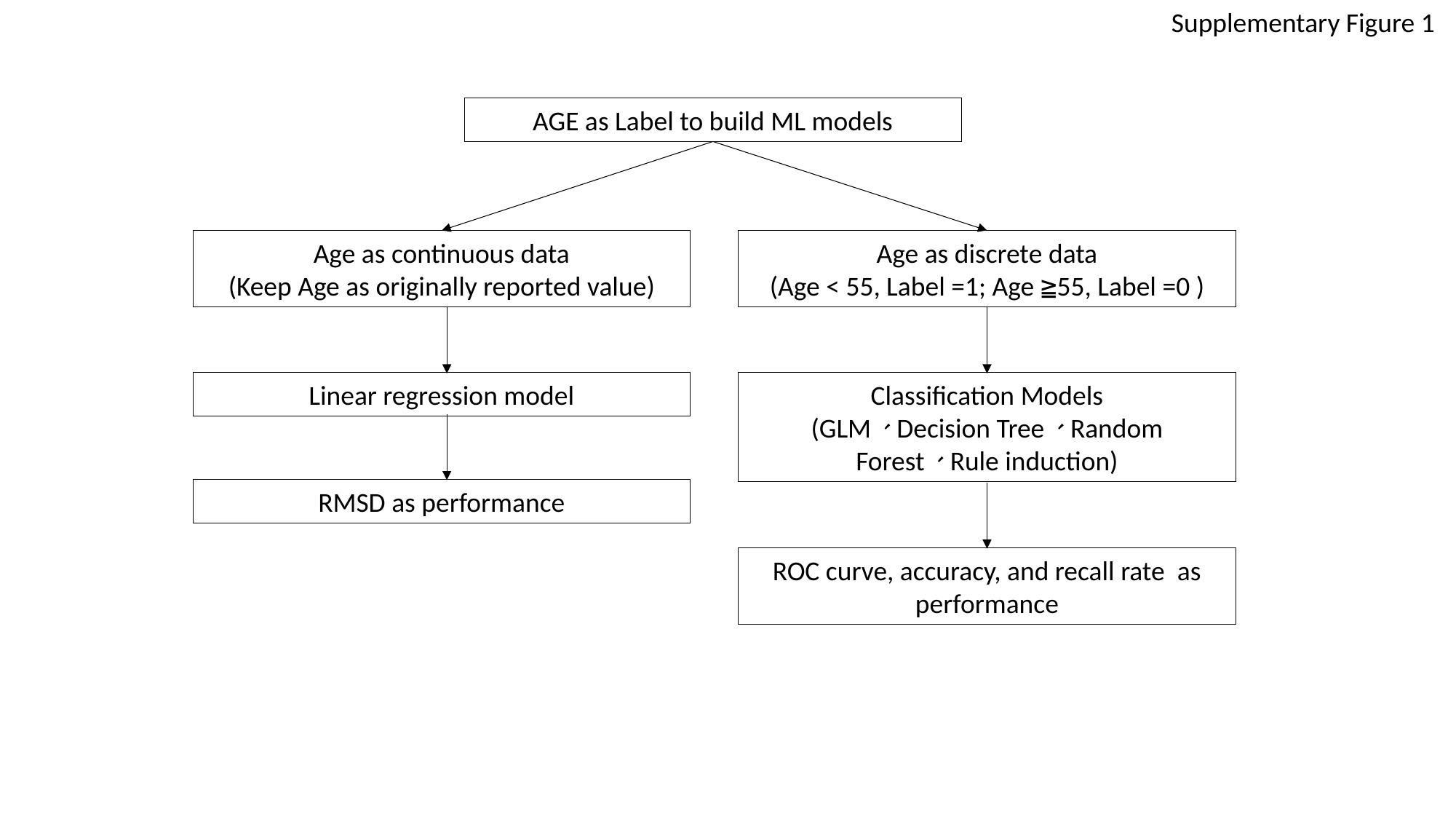

Supplementary Figure 1
AGE as Label to build ML models
Age as continuous data
(Keep Age as originally reported value)
Linear regression model
RMSD as performance
Age as discrete data
(Age < 55, Label =1; Age ≧55, Label =0 )
Classification Models
(GLM、Decision Tree、Random Forest、Rule induction)
ROC curve, accuracy, and recall rate as performance

## Slide 2
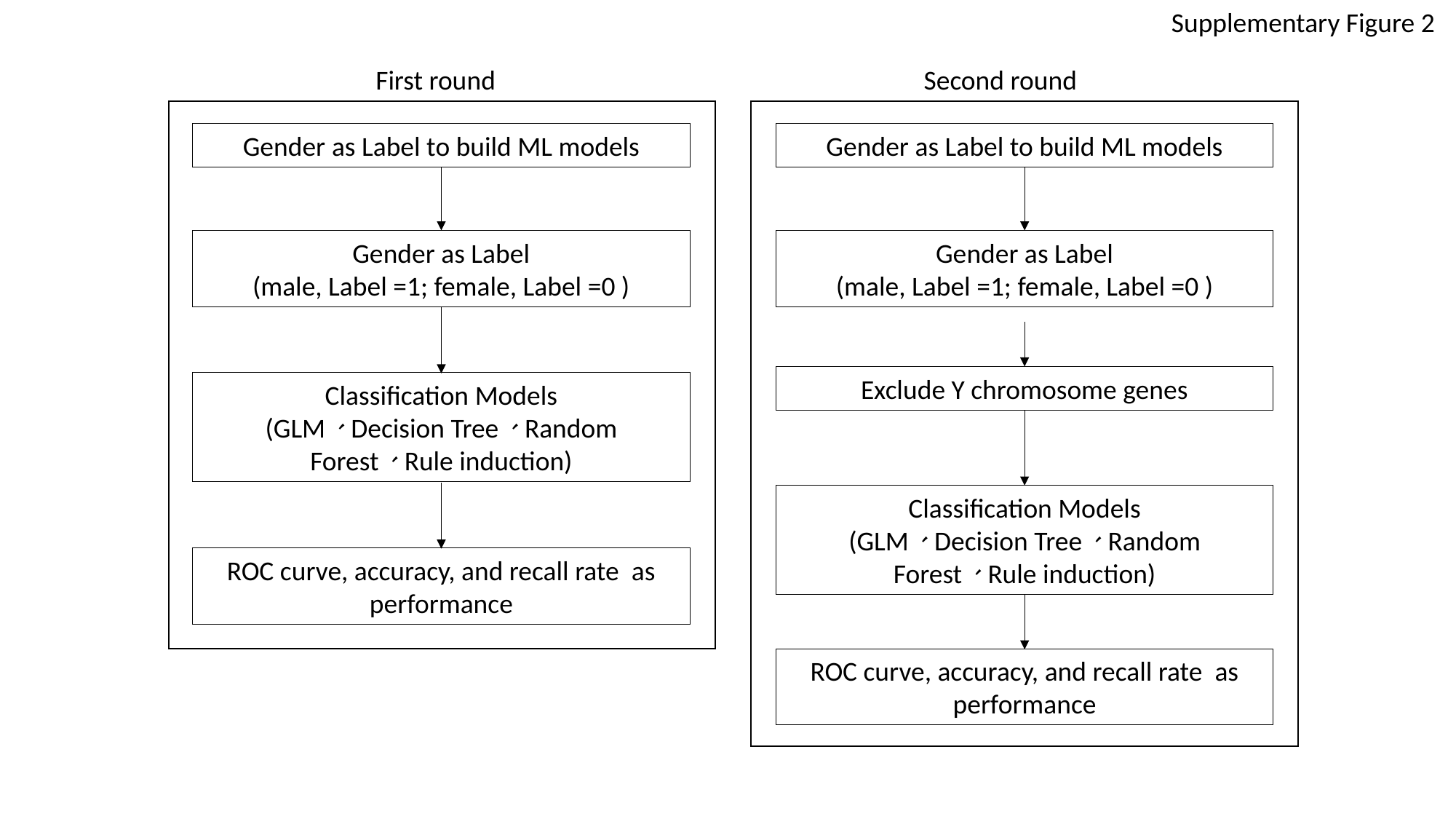

Supplementary Figure 2
First round
Second round
Gender as Label to build ML models
Gender as Label to build ML models
Gender as Label
(male, Label =1; female, Label =0 )
Gender as Label
(male, Label =1; female, Label =0 )
Exclude Y chromosome genes
Classification Models
(GLM、Decision Tree、Random Forest、Rule induction)
Classification Models
(GLM、Decision Tree、Random Forest、Rule induction)
ROC curve, accuracy, and recall rate as performance
ROC curve, accuracy, and recall rate as performance

## Slide 3
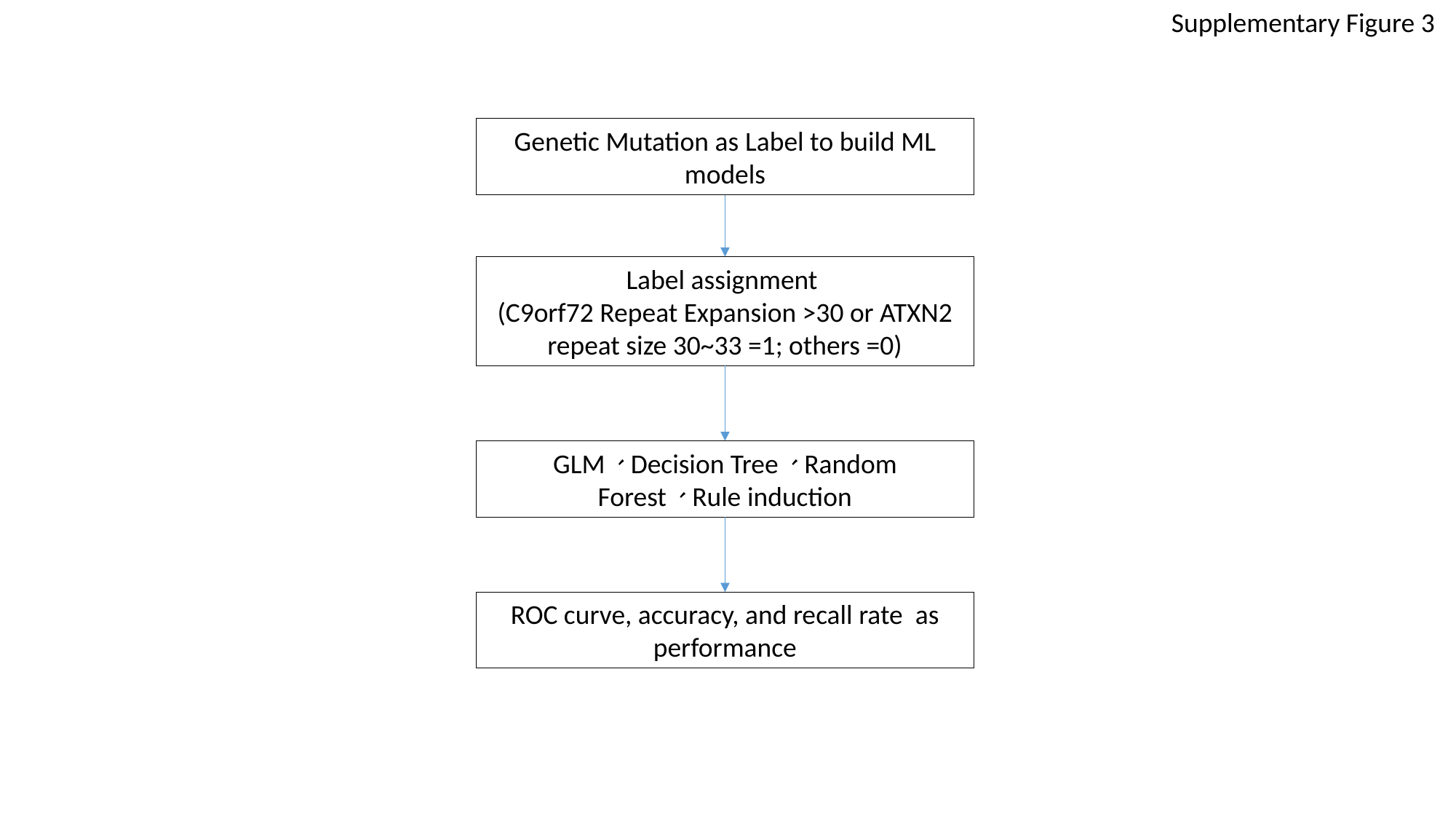

Supplementary Figure 3
Genetic Mutation as Label to build ML models
Label assignment
(C9orf72 Repeat Expansion >30 or ATXN2 repeat size 30~33 =1; others =0)
GLM、Decision Tree、Random Forest、Rule induction
ROC curve, accuracy, and recall rate as performance
