# Supplementary material for: Machine learning identified novel players in lipid metabolism, endosomal trafficking, and iron metabolism of the ALS spinal cord
Source: Sci Rep. 2025 Jan 10;15:1564. doi: 10.1038/s41598-024-81315-z (PMC11723943; doi:10.1038/s41598-024-81315-z)
Supplement: Supplementary file 3 — Supplementary Information 3. [file 41598_2024_81315_MOESM3_ESM.docx]

**Machine learning identified novel players in lipid metabolism, endosomal trafficking, and iron metabolism of the ALS spinal cord**

Jack Cheng ^1,2,a^, Bor-Tsang Wu ^3,a^, Hsin-Ping Liu ^4*^, Wei-Yong Lin ^1,2*^

^1^ Graduate Institute of Integrated Medicine, College of Chinese Medicine, China Medical University, Taichung 40402, Taiwan

^2^ Department of Medical Research, China Medical University Hospital, Taichung 40447, Taiwan

^3^ Department of Senior Citizen Service Management, National Taichung University of Science and Technology, Taichung City, 40343, Taiwan

^4^ Graduate Institute of Acupuncture Science, College of Chinese Medicine, China Medical University, Taichung 40402, Taiwan

^a^ These authors contributed equally to this work.

***** Correspondence: linwy@mail.cmu.edu.tw (W.-Y.L.) ; hpliu@mail.cmu.edu.tw (H.-P.L.)

**ORCID:**

Jack Cheng 0000-0002-9305-0781

Hsin-Ping Liu 0000-0002-2569-3072

Wei-Yong Lin 0000-0002-8443-6180

**Supplementary Tables & Figures**

**Supplementary Table 1.** Generalized linear model build by machine learning using NGS data of Spinal Cord Cervical from CTRL and ALS samples.

**Supplementary Table 2.** Identified genes by machine learning using NGS data of Spinal Cord Cervical from CTRL and ALS samples.

**Supplementary Table 3.** NGS data of Spinal Cord Cervical from CTRL and ALS samples.

**Supplementary Table 4.** Supplementary Table 4. NGS data of Spinal Cord Lumbar from CTRL and ALS samples.

**Supplementary Table 5.** Clinical information of ALS samples.

**Supplementary Table 6.** Generalized linear model build by machine learning to predict gender of ALS samples.

**Supplementary Figure 1.** Workflow of building ML models with age of ALS samples as the label of models.

**Supplementary Figure 2.** Workflow of building ML models with gender of ALS samples as the label of models.

**Supplementary Figure 3.** Workflow of building ML models with genetic mutation of ALS samples as the label of models.
